# Doses of Quercetin in the Range of Serum Concentrations Exert Delipidating Effects in 3T3-L1 Preadipocytes by Acting on Different Stages of Adipogenesis, but Not in Mature Adipocytes

**DOI:** 10.1155/2015/480943

**Published:** 2015-06-09

**Authors:** Itziar Eseberri, Jonatan Miranda, Arrate Lasa, Itziar Churruca, María P. Portillo

**Affiliations:** ^1^Nutrition and Obesity Group, Department of Nutrition and Food Science, Faculty of Pharmacy and Lucio Lascaray Research Institute, University of País Vasco (UPV/EHU), 01006 Vitoria, Spain; ^2^CIBEROBN Physiopathology of Obesity and Nutrition, Institute of Health Carlos III (ISCIII), 01006 Vitoria, Spain

## Abstract

*Scope*. To determine whether doses of quercetin in the range of serum concentrations exert any effect on triacylglycerol accumulation in maturing preadipocytes and mature adipocytes. The influence on the expression of adipogenic markers as well as on gene expression and activity of enzymes involved in triacylglycerol metabolism were assessed. *Methods and Results*. 3T3-L1 preadipocytes were treated during differentiation and mature adipocytes for 24 hours with low doses (0.1–10 *µ*M) of quercetin. Triacylglycerol content in both cell types and free fatty acid and glycerol in the incubation medium of mature adipocytes were measured spectrophotometrically. Gene and protein expression were assessed by RT-PCR and Western blot. LPL and FAS activities were quantified. During differentiation quercetin reduced triacylglycerol content at doses from 0.5 to 10 *µ*M. 1 *µ*M of quercetin reduced C/EBP*β* gene expression, SREBP1 mature protein levels, and PPAR*γ* gene expression. 10 *µ*M of quercetin reduced LPL gene expression and PPAR*γ* and SREBP1c expression. In mature adipocytes, only 10 *µ*M of quercetin reduced triacylglycerol content. Lipogenic FAS expression and activity were reduced at this dose. *Conclusion*. Quercetin, in the range of serum concentrations, is able to inhibit adipogenesis, but higher doses, at least 10 *µ*M, are needed to reduce fat accumulation in mature adipocytes.

## 1. Introduction

Overweight and obesity have become a public health problem in developed societies due to their high prevalence [1–3]. They cause numerous metabolic alterations and comorbidities such as insulin resistance, diabetes, dyslipidemia, and hypertension [4, 5]. Scientific research is constantly looking for new molecules which could be used as effective functional biomolecules in the fight against this disease and its comorbidities.

Among these molecules, flavonoids, a group of natural substances which have a variable phenolic structure and are found in fruits, vegetables, tea, and wine, have received a great deal of interest in recent years because they have been reported to have beneficial effects on health [6–9]. Quercetin, the most abundant flavonoid [10], is present in onions, broccoli, tomatoes, apples, and berries [11]. Its intake in the diet is higher than that of other polyphenols [12]. A wide range of biological effects, such as prevention of oxidation, inflammation, and cancer, have been attributed to this compound [13–16]. It has also been reported to improve diabetic status in animal models of either type 1 or type 2 diabetes [17]. With regard to obesity, data concerning its delipidating effect, as well as its mechanisms of action, are scarce.

In this context, the present study aimed to analyze the effect of low doses of quercetin on triacylglycerol accumulation in both maturing preadipocytes and mature adipocytes. In order to gain insight into the mechanisms underlying this action, the influence on the expression of adipogenic markers, as well as on gene expression, and activity of enzymes involved in triacylglycerol metabolism were assessed.

It is important to underline that our main interest focused on concentrations of this polyphenol which were lower than those used in the reported studies (10 to 500 *μ*M) [18–24], which are far from those reached by this molecule in plasma after oral ingestion [25–27].

## 2. Materials and Methods

### 2.1. Reagents

Dulbecco's modified Eagle's medium (DMEM) was purchased from GIBCO (BRL Life Technologies, Grand Island, NY). Quercetin was purchased from Sigma (St. Louis, MO, USA). Triacylglycerols (TG) were determined by Infinity Triglycerides reagent (Thermo Electron Corporation, Rockford, IL, USA) and protein concentrations of cell extracts were measured with BCA reagent (Thermo Scientific, Rockford, IL, USA). Commercial kits for analyzing free fatty acids and free glycerol were supplied by Roche and Sigma, respectively (Free Fatty Acids, Half Micro Test, Roche, Basilea, Sweden, and F6428, Sigma, St. Louis, MO, USA).

### 2.2. Experimental Design

3T3-L1 preadipocytes, supplied by American Type Culture Collection (Manassas, VA, USA), were cultured in DMEM containing 10% foetal calf serum (FCS). Two days after confluence (day 0), the cells were stimulated to differentiate with DMEM containing 10% FCS, 10 *μ*g/mL insulin, 0.5 mM isobutylmethylxanthine (IBMX), and 1 *μ*M of dexamethasone for 2 days. From day 4 onward, the differentiation medium was replaced by FBS/DMEM medium (10%) containing 0.2 *μ*g/mL insulin. This medium was changed every two days until cells were harvested. All media contained 1% penicillin/streptomycin (10,000 U/mL), and the media for differentiation and maturation contained 1% (v/v) of biotin and pantothenic acid. Cells were maintained at 37°C in a humidified 5% CO_2_ atmosphere.

### 2.3. Cell Treatment

For the treatment of maturing preadipocytes, cells grown in 6-well plates were incubated with quercetin, at 0.1, 0.5, 1, 2, 5, and 10 *μ*M (diluted in 95% ethanol), during differentiation. In the case of the control group the same volume of the vehicle (ethanol 95%) was used. Media containing or not containing quercetin were changed every two days: on day 0, day 2, day 4, and day 6. On day 8, supernatant was collected and cells were used for TG determination and RNA extraction. Each experiment was performed 3 times.

In order to distinguish the effects of quercetin in early and late stages of adipogenesis additional cultures were carried out. To assess the effects on early stages, cells grown in 6-well plates were incubated with quercetin at doses of 1, 2, 5, and 10 *μ*M (diluted in 95% ethanol) from day 0 to 60 hours after the induction of differentiation. The effects on late stages were assessed by incubating cells grown in 6-well plates, with the same doses of quercetin, from 60 hours after differentiation to day 8, as reported by Tang et al. [28]. In the case of the control group the same volume of the vehicle (ethanol 95%) was used. Incubation media containing or not containing quercetin were changed every two days. Supernatants were collected and cells were used for TG determination. Each experiment was performed 3 times.

For the treatment of mature adipocytes, cells grown in 6-well plates were incubated with quercetin, at 1, 2, 5, and 10 *μ*M (diluted in 95% ethanol), on day 12 after differentiation (>90% of cells showed visible lipid droplets). In the case of the control group the same volume of the vehicle (ethanol 95%) was used. After 24 hours, supernatant was collected and cells were used for TG determination and RNA extraction. Each experiment was performed 3 times.

### 2.4. Measurement of Triacylglycerol Content in Adipocytes

After treatment, the medium was removed and cell extracts were used for TG determination. Maturing preadipocytes and mature adipocytes were washed extensively with phosphate-buffered saline (PBS) and incubated 3 times with 800 *μ*L of hexane/isopropanol (2 : 1). The total volume was then evaporated by vacuumed centrifugation and the pellet was resuspended in 200 *μ*L Tritón X-100 in 1% distilled water. Afterwards, TGs were disrupted by sonication and the content was measured by means of a commercial kit. For protein determinations, cells were lysed in 0.3 N NaOH, 0.1% SDS. Protein measurements were performed using the BCA reagent. TG content values were obtained as mg triacylglycerols/mg protein and converted into arbitrary units.

### 2.5. RNA Preparation and Quantitative Real Time PCR

RNA samples from cells treated were extracted using Trizol (Invitrogen, Carlsbad, CA, USA), according to the manufacturer's instructions. The integrity of the RNA extracted from all samples was verified and quantified using a RNA 6000 Nano Assay (Thermo Scientific, Wilmington, DE, USA). RNA samples were then treated with DNase I kit (Applied Biosystems, California, USA) to remove any contamination with genomic DNA.

1.5 *μ*g of total RNA of each sample was reverse-transcribed to first-strand complementary DNA (cDNA) using iScript cDNA Synthesis Kit (Bio-Rad, Hercules, CA, USA). Relative CCAAT-enhancer-binding proteins *α* and *β* (C/EBP*α* and C/EBP*β*), peroxisome proliferator-activated receptor *γ* (PPAR*γ*), lipoprotein lipase (LPL), and sterol regulatory element-binding factor 1c (SREBF1C) mRNA levels in maturing preadipocytes and relative adipose triglyceride lipase (ATGL), hormone sensitive lipase (HSL), lipoprotein lipase (LPL), fatty acid synthase (FASN), acetyl CoA carboxylase (ACC), deacetylase sirtuin 1 (SIRT1), leptin, adiponectin, visfatin, and apelin mRNA levels in mature adipocytes were quantified using real time PCR with an iCycler-MyiQ Real Time PCR Detection System (BioRad, Hercules, CA, USA). *β*-actin mRNA levels were similarly measured and served as the reference gene. The PCR reagent mixture consisted of 4.75 *μ*L aliquot of each diluted cDNA, SYBR Green Master Mix (Applied Biosystems, Foster City, CA, USA), and the upstream and downstream primers (300 nM each, except in the case of CEBP*α* and apelin, whose primer concentration was 600 nM, and ATGL whose primer concentration was 900 nM). In the case of CEBP *β*, SIRT-1, and visfatin the reagent mixture consisted of 1 *μ*L of each cDNA, Premix Ex TaqTM (Takara, USA), and the upstream and downstream primers (600 nM for CEBP *β*, visfatin, and *β*-actin and 900 nM for SIRT-1). Specific primers and probes were synthesized commercially (Tib Molbiol, Berlin, Germany, and Eurogentec, Liège, Belgium) (Table 1).

RT-PCR parameters were as follows: initial 2 min at 50°C, denaturation at 95°C for 10 min followed by 40 cycles of denaturation at 95°C for 30 s, annealing at 60°C for 30 s (except in the case of LPL, PPAR*γ*, and CEBP*α* in maturing preadipocytes where the annealing was at 62.1°C, 63.9°C, and 66.4°C, resp., and leptin in mature adipocytes where the annealing was at 63.9°C and 66.4°C, resp.), and extension at 60°C for 30 s. All sample mRNA levels were normalized to the values of *β*-actin and the results expressed as fold changes of threshold cycle (Ct) value relative to controls using the 2^−ΔΔCt^ method [29].

### 2.6. Western Blot Analysis

Total proteins were isolated from maturing 3T3-L1 adipocytes using the modified Trizol (Invitrogen, Carlsbad, CA, USA) method [30], and the protein concentration was determined by BCA assay (Pierce, USA). Total protein (70 *μ*g) was subjected to 7.5% SDS-polyacrylamide gel, electroblotted onto PVDF membranes (Millipore, Bradford, MA, USA), and immunodetected using mouse anti-SREBP1 (1 : 1000), mouse anti-PPAR*γ* (1 : 1000), mouse anti-*β*-actin (1 : 5000) (Santa-Cruz Biotech, CA, USA), and goat anti-mouse immunoglobulin G-horseradish peroxidase conjugate (1 : 5000) (Santa-Cruz Biotech, CA, USA) with ChemiDoc MP imaging system (BioRad, CA, USA).

### 2.7. Enzymatic Activity

Lipoprotein lipase enzyme activity was assessed following the method described by del Prado et al. [31] with modifications. For total lipoprotein lipase (LPL) activity determination, 130 *μ*g of protein homogenate (0.3 N NaOH, 0.1% SDS) was incubated (15 min, 37°C) with a 2.9 mL of a buffer containing 1.5 mL of dibutyryl fluorescein (20 *μ*M), 150 *μ*L of 2-methoxyethanol, and 1.25 mL of phosphate buffer (3 mM NaH_2_PO_4_ and 50 mM Na_2_HPO_4_, with or without 2.5 M NaCl). Subsequently the reaction was halted in ice. Finally, fluorescence was measured. Total LPL activity was calculated by subtracting non-LPL lipolytic activity in the presence of NaCl from the total lipolytic activity, determined without NaCl, and expressed as* p*mol oleate released per minute per *μ*g of protein.

FAS activity was measured by a spectrophotometer at 340 nm of NADPH absorption. The overall reaction system contained 100 *μ*L of 1 M KH_2_PO_4_, 50 *μ*L of 50 mM EDTA, 100 *μ*L of cysteine 100 mM, 50 *μ*L of bovine serum albumin 6 mg/mL, 50 *μ*L of 1.2 mM acetyl-CoA, 20 *μ*L of 10 *μ*M malonyl-CoA, 65 *μ*L of 2.4 mM NADPH, and 150 *μ*g of protein homogenate (0.3 N NaOH, 0.1% SDS pH 7.4) in a total volume of 1.75 mL as previously described [32].

### 2.8. Measurements of Glycerol and Free Fatty Acids in the Media

After treatment in mature adipocytes, aliquots of the medium treated with 10 *μ*M of quercetin were removed and analyzed for glycerol and free fatty acid (FFA) quantification by using commercial kits. Results were expressed as *μ*g glycerol/mg protein and nmol FFA/mg protein.

### 2.9. Statistical Analysis

Results are presented as mean ± standard error of the mean. Statistical analysis was performed using SPSS 20.0 (SPSS Inc. Chicago, IL, USA). Comparisons between each treatment and the controls were analyzed by Student's *t*-test. Statistical significance was set up at the *P* < 0.05 level.

## 3. Results

### 3.1. Effect of Quercetin on Triacylglycerol Content in Maturing Preadipocytes

When cells were treated with quercetin from day 0 to day 8 (whole adipogenesis), triacylglycerol content was significantly reduced by all the doses used, with the exception of 0.1 *μ*M which only showed a tendency towards lower values (Table 2). The greatest percentages of reduction in triacylglycerol content were obtained with 1 and 10 *μ*M (−42% and −37%, resp.). When cultures were carried out at early and late stages by using these doses, it was observed that 1 *μ*M of quercetin tended to reduce TG accumulation during the early stages of adipogenesis, but not during the late stages (Table 2). By contrast, 10 *μ*M of quercetin was only able to decrease TG content in the late stage of differentiation (Table 2).

### 3.2. Effect of Quercetin on Adipogenic Gene Expression

**Figure 1:**
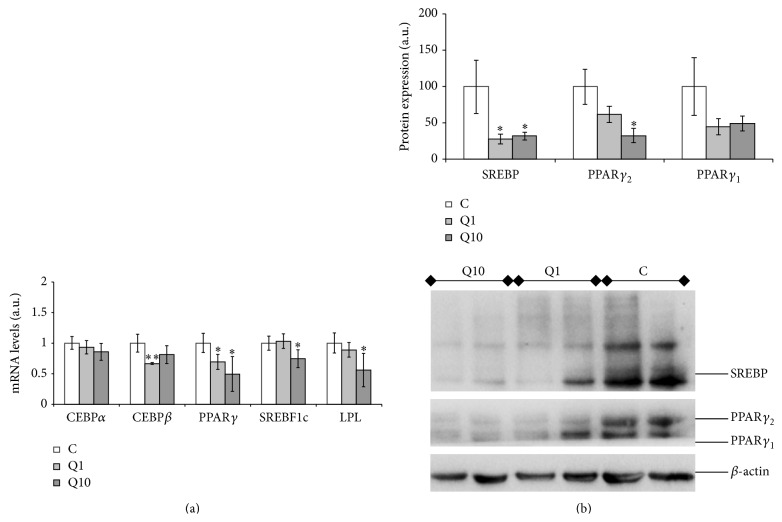
Effects of 1 and 10 *μ*M of quercetin (Q1 and Q10) on gene expression of CEBP*β*, CEBP*α*, PPAR*γ*, SREBF1c, and LPL (a) and on protein expression of PPAR*γ*_1_, PPAR*γ*_2_, and SREBP1 (b) in 3T3-L1 maturing preadipocytes treated from day 0 to day 8. Values are means ± SEM. Comparisons between each treatment and the controls were analyzed by Student's *t*-test. The asterisks represent differences versus the controls (^*^ *P* < 0.05; ^**^ *P* < 0.01).

For this purpose, 1 and 10 *μ*M of quercetin were selected because they induced the greatest delipidating effects. The dose of 1 *μ*M significantly reduced C/EBP*β* and PPAR*γ* expression (Figure 1(a)). The dose of 10 *μ*M significantly reduced the expression of PPAR*γ*, SREBF1c, and LPL (Figure 1(a)).

### 3.3. Effect of Quercetin on Adipogenic Protein Expression

The dose of 1 *μ*M of quercetin did not modify the protein levels of PPAR*γ*_1_ and PPAR*γ*_2_, but it did induce a decrease in protein expression of mature SREBP1 (68 kDa) (Figure 1(b)). The dose of 10 *μ*M significantly reduced protein expression of mature SREBP1 and PPAR*γ*_2_, but not that of PPAR*γ*_1_ (Figure 1(b)).

### 3.4. Effect of Quercetin on Triacylglycerol Content in Mature Adipocytes

When mature adipocytes were treated with 1 *μ*M of quercetin no effects on TG content were observed. The doses of 2 and 5 *μ*M induced reductions that did not reach statistical significance, and only the highest dose (10 *μ*M) showed a significant effect (Table 2).

### 3.5. Effect of Quercetin on the Expression of Genes Involved in Triacylglycerol Metabolism in Mature Adipocytes

**Figure 2:**
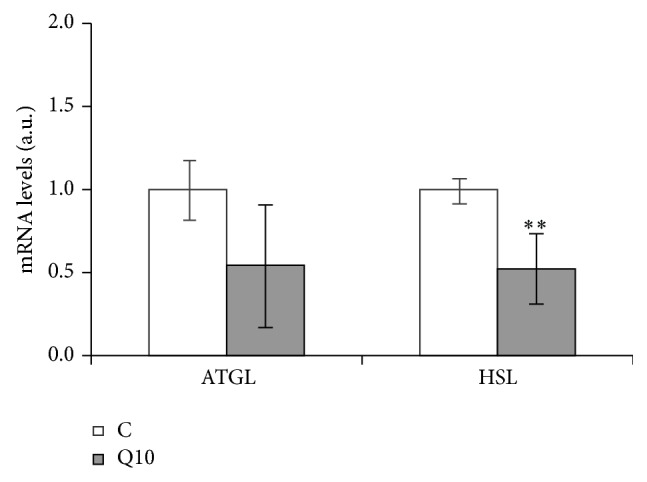
Effects of 10 *μ*M of quercetin (Q10) on the gene expression of lipases, ATGL and HSL, in 3T3-L1 mature adipocytes treated for 24 h. Values are means ± SEM. Comparisons between each treatment and the controls were analyzed by Student's *t*-test. The asterisks represent differences versus the controls (^**^ *P* < 0.01).

**Figure 3:**
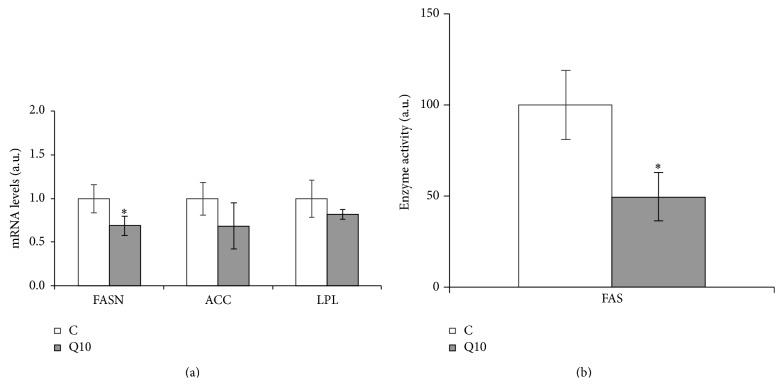
Effects of 10 *μ*M of quercetin (Q10) on the gene expression of FASN, ACC, and LPL (a) and on the activity of FAS enzyme (b), in 3T3-L1 mature adipocytes treated for 24 h. Values are means ± SEM. Comparisons between each treatment and the controls were analyzed by Student's *t*-test. The asterisks represent differences versus the controls (^*^ *P* < 0.05).

In mature adipocytes, gene expression was analyzed only in cells treated with 10 *μ*M of quercetin because this dose was the only effective one. With regard to lipolytic enzymes, HSL expression was significantly reduced and ATGL remained unchanged (Figure 2). FASN expression was significantly reduced, but no changes were observed in ACC and LPL (Figure 3(a)).

**Figure 4:**
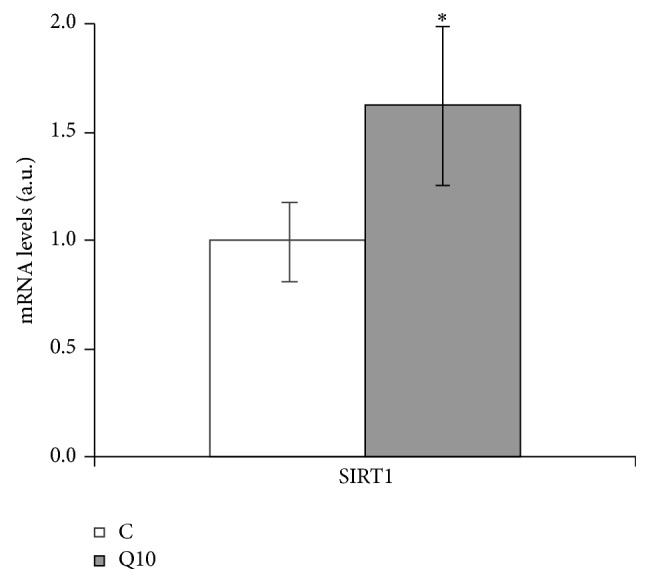
Effects of 10 *μ*M of quercetin (Q10) on the gene expression of SIRT1 in 3T3-L1 mature adipocytes treated for 24 h. Values are means ± SEM. Comparisons between each treatment and the controls were analyzed by Student's *t*-test. The asterisk represents differences versus the controls (^*^ *P* < 0.05).

When SIRT1 expression was measured, 10 *μ*M of quercetin increased SIRT1 mRNA levels (Figure 4).

### 3.6. Effect of Quercetin on Lipoprotein Lipase and Fatty Acid Synthase Activities in Mature Adipocytes

In order to find potential changes in LPL at a posttranscriptional level, the activity of this enzyme was measured in mature cells treated with 10 *μ*M of quercetin and no changes were observed (0.90 ± 0.14* p*mol fluorescein/*μ*g protein/min in the case of the control group and 1.15 ± 0.23* p*mol oleate/*μ*g protein/min in the case of the group treated with quercetin at 10 *μ*M; *P* = 0.38).

The dose of 10 *μ*M of quercetin had an inhibitory effect on FAS activity. This result is in line with the reduced gene expression of this enzyme (Figure 3(b)).

### 3.7. Effect of Quercetin on Glycerol and Free Fatty Acid Release in Mature Adipocytes

In order to know if the decrease in HSL expression was accompanied by changes in the lipolytic pathway, glycerol and free fatty acids were quantified in the incubation media, as markers of lipolysis. Both parameters remained unchanged after treatment of cells with 10 *μ*M of quercetin (65.3 ± 7.5 versus 60.6 ± 14.9 nmol FFA/mg protein and 393.05 ± 10.1 versus 429.16 ± 31.6 *μ*g glycerol/mg protein for control and 10 *μ*M quercetin-treated cells, resp.).

### 3.8. Effect of Quercetin on Adipokine Gene Expression in Mature Adipocytes

**Figure 5:**
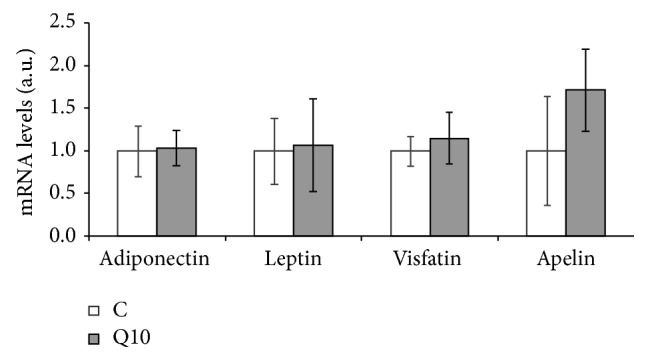
Effects of 10 *μ*M of quercetin (Q10) on the gene expression of adiponectin, leptin, visfatin, and apelin in 3T3-L1 mature adipocytes treated for 24 h. Values are means ± SEM. Comparisons between each treatment and the controls were analyzed by Student's *t*-test.

No changes were observed in gene expression of adiponectin, leptin, visfatin, and apelin in mature adipocytes treated with 10 *μ*M of quercetin (Figure 5).

## 4. Discussion

In recent years, a great number of studies have been conducted in the field of natural compounds in order to find new tools to combat obesity. In this line, one of the most studied molecules has been resveratrol, a polyphenol (stilbene) present in grapes and wine, which has shown a clear antiobesity effect in cultured cells [33–41] and in animal models [40, 42–48]. The effects of flavonoids, which show interesting biological effects on cancer and diabetes [7, 9], on obesity prevention or treatment have not been so widely analyzed [49]. The present study focuses on quercetin, the most abundant flavonoid in food stuffs [11].

To date, there are only few studies in the literature demonstrating that quercetin reduces triacylglycerol accumulation in cultured adipocytes and animal models, and very little has been reported concerning the mechanisms of action [18–23, 50]. One advantage of* in vitro* studies is that they allow one to clearly differentiate between the effects of a molecule on preadipocytes (and thus on adipogenesis) and on mature adipocytes. This is the reason for choosing this experimental model for our study.

It is important to point out that previously reported* in vitro* studies have been performed by using high doses of quercetin (10 to 500 *μ*M). These doses are far from those achieved in plasma in humans and animals. Thus, in the present study we aimed to complete the information provided in the abovementioned studies by analyzing the effects of quercetin on cultured adipocytes at lower doses. In fact doses from 0.1 to 2 *μ*M are in the range of quercetin concentrations found in plasma in several studies performed either in rodents or in humans [26, 27, 51, 52].

Quercetin has been demonstrated to exert genotoxicity and mutagenicity in* in vitro* experiments. Nevertheless, according to the critical examination of quercetin safety carried out by Harwood et al. [27], 34 *μ*M was the lowest dose to show this effect in mouse cells, specifically in mouse lymphoma L5178Y cells [53]. Thus, we assumed that the doses used in the present study did not exert this toxic effect.

All the quercetin doses tested in the present study (0.1–10 *μ*M) significantly reduced TG content in 3T3-L1 maturing preadipocytes, with the exception of 0.1 *μ*M which only showed a tendency towards lower values (8 days of treatment after the confluence). In the experiment reported by Ahn et al. [20] in 3T3-L1 preadipocytes, cells were treated with 10, 50, and 100 *μ*M of quercetin during differentiation. The three doses of this flavonoid reduced TG content. Data concerning 10 *μ*M of quercetin are in good accordance with the present study. Yang et al. [21] used 12.5 *μ*M and 25 *μ*M of quercetin for 3T3-L1 preadipocyte incubation, and only the highest dose was effective in TG reduction. The difference between the present study and that reported by Yang et al. could be the treatment duration (from day 0 to day 8 in the present study and from day 0 to day 6 in the study reported by Yang et al.). The effect of 5 *μ*M of quercetin is also in the same line as that reported by Bae et al. (2014) [54] by using low doses of quercetin (3.3–6.6 *μ*M). With regard to the lowest doses used in the present study (0.1, 0.5, 1, and 2 *μ*M), as far as we know, this is the first time that they have been used and thus comparisons with the literature cannot be made.

To compare the delipidating effect of quercetin during adipogenesis with that observed with other polyphenols is a matter of interest. In a recent study performed in our laboratory we analyzed the delipidating effect of resveratrol at 1, 10, and 25 *μ*M, in maturing preadipocytes by using the same experimental conditions [37]. No changes were observed in TG content at 1 and 10 *μ*M, but the polyphenol was effective at 25 *μ*M. These results show that quercetin is more efficient in reducing adipogenesis than resveratrol is. There are very few studies where low concentrations of other flavonoids have been tested in 3T3-L1 maturing preadipocytes. Yang et al. showed that 3.125, 6.25, and 12.5 *μ*M of xanthohumol and isoxanthohumol, flavonoids present in hops, reduced TG accumulation during adipocyte differentiation [55]. Nevertheless, other authors have not found this antiadipogenic effect in the case of other flavonoids (naringenin) or have stated that higher doses are needed to reach this effect (genistein) [22, 56, 57].

**Figure 6:**
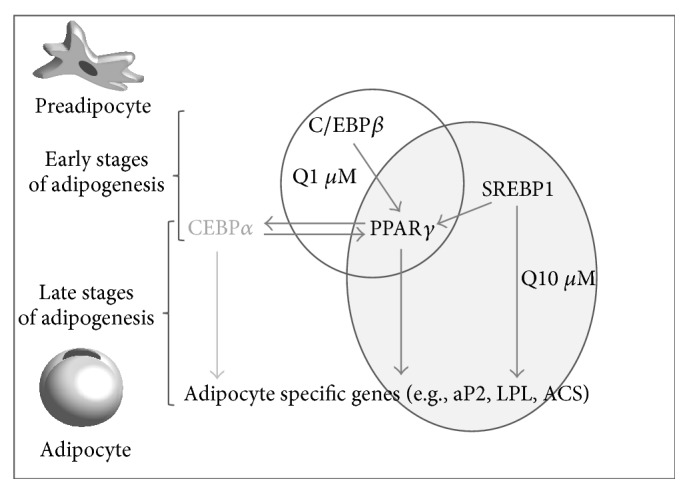
Effects of different doses of quercetin in the pathways of the adipogenic process.

Two phases can be distinguished in adipogenesis, the premitotic phase (early stage of differentiation; 60 hours after confluence) [28] and the postmitotic phase (late stage). During the early phase C/EBP*β* and SREBP1c expressions are increased. This change, in turn, triggers high level expression of PPAR*γ*, which is considered the master coordinator of adipocyte differentiation. C/EBP*α* and LPL are induced during later stage of differentiation [58]. Interestingly, when the influence of quercetin on the expression of these genes was assessed it was observed that, depending on the dose, the mechanisms of action of this flavonoid to reduce adipogenesis were different (Figure 6). Quercetin, at a dose of 1 *μ*M, significantly reduced C/EBP*β* and PPAR*γ* gene expressions, but PPAR*γ*_1_ and PPAR*γ*_2_ protein levels remained unchanged. Moreover, a reduction in protein expression of mature SREBP1c was observed. As stated before, C/EBP*β* and SREBP1c are adipogenic markers expressed in the early stage of the process [28]. Taken together these results suggest that adipogenesis was stopped at this phase. This fact was confirmed by the reduction in TG content (tendency) observed after 60 hours of treatment, but not when preadipocytes were treated from 60 hours to 8 days.

Treatment with 10 *μ*M of quercetin reduced both gene and protein expressions of PPAR*γ* and SREBP1c, as well as LPL gene expression. These results are in good accordance with those reported by other authors [20, 21] who showed that 10 and 25 *μ*M of quercetin reduced PPAR*γ* and SREBP1c expression. Although SREBP1c expression starts at early stages of differentiation, protein expression of mature SREBP reaches its peak at the 5th-6th day after the confluence [59]. For this reason SREBP1c is also considered an important factor in the late stages of adipogenesis, as is the case of PPAR*γ*. The reduction in PPAR*γ* and SREBP1c expression after treatment with 10 *μ*M of quercetin, together with the strong decrease in TG content observed when maturing adipocytes were treated with this dose of quercetin from 60 hours to 8th day after the confluence, suggests that this dose acts primarily at the late stage of adipogenesis.

When the effects on mature adipocytes were assessed, quercetin significantly reduced TGs at the dose of 10 *μ*M. These results are in the same line as those obtained by Park et al. by incubating murine adipocytes, but with a higher dose (25 *μ*M) of this flavonoid [22]. In our previous study, where mature adipocytes were treated with 1, 10, and 25 *μ*M of resveratrol during 24 hours [37], the lowest dose of this polyphenol resulted in a significant reduction in TG content. By comparing the present data with those of this study, it can be suggested that quercetin is less efficient than resveratrol when delipidating mature adipocytes. The effect of other flavonoids at low doses on mature adipocytes has not been widely analyzed. Reported studies show that doses around 100 *μ*M are needed in the case of naringenin and genistein to reach a reduction in TG content [56].

The expression of genes involved in TG metabolism in mature adipocytes was analyzed by real time RT-PCR after 24 hours of treatment. Quercetin reduced HSL expression but did not change that of ATGL. Taking into account that HSL is an enzyme regulated mainly at posttranscriptional level and considering that a reduction in the expression of a lipolytic enzyme is not an expected result for a delipidating molecule, the release of glycerol and free fatty acids to the incubation medium was quantified as an index of lipolytic activity. No changes were observed in treated cells when compared with the controls, and thus a lack of effect of quercetin in HSL activity could be suggested, despite its downregulation.

Gene expression of fatty acid synthase, as well as the activity of this enzyme, was lower after quercetin treatment, suggesting that the delipidating effect of this molecule could be due to its inhibitory effect on* de novo* lipogenesis. When the expression of LPL, the enzyme which allows adipose tissue to uptake free fatty acids from TGs circulating in lipoproteins, was measured, no changes were observed in quercetin-treated mature adipocytes. This is a surprising result because as quercetin reduced LPL mRNA expression in maturing preadipocytes, a similar effect could be expected in mature adipocytes. Nevertheless, it is important to highlight that LPL is controlled not only transcriptionally but also posttranscriptionally [60]. Given this, in order to find potential changes in LPL at a posttranscriptional level the activity of this enzyme was measured in mature adipocytes treated with 10 *μ*M of quercetin. No changes were observed and thus it may be suggested that the metabolic process controlled by this enzyme was not affected by the flavonoid.

In previous studies performed in our laboratory, incubation with 10 *μ*M resveratrol increased ATGL expression and reduced FASN gene expression [37, 41]. These data suggest that while 10 *μ*M of quercetin exerts its delipidating effect via inhibition of adipogenesis and lipogenesis, resveratrol acts by increasing lipolysis and inhibiting lipogenesis.

It has been reported that several beneficial effects of polyphenols are mediated by the deacetylase SIRT1 [61]. In the present study the incubation of mature adipocytes with 10 *μ*M of quercetin led to increased SIRT1 expression. SIRT1 can induce SREBP1c deacetylation, which leads to the inactivation of this transcription factor and thus to decreased expression of lipogenic enzymes [62]. Taking this into account, it can be proposed that the increased expression of SIRT1 observed in mature adipocytes in the present study could be related to the reduced expression of FASN.

In relation to glycaemic control, flavonoids have been reported to improve insulin sensitivity by modifying adipokines secretion. Specifically, quercetin has been demonstrated to inhibit visfatin secretion in SGBS human adipocytes or to increase adiponectin secretion in rats fed a high fat diet [23, 63]. As far as we know, the effect of low doses of quercetin on adipokine expression and secretion has not been analyzed to date. In the present study no changes were observed in adipokine gene expression, suggesting that low doses of this flavonoid do not affect these mediators of insulin signalling and glycaemic control.

It has been reported that quercetin is able to reduce resveratrol metabolism; more specifically it can decrease the formation of sulfate metabolites [64]. Thus, the combination of quercetin and resveratrol has been proposed as a tool to increase the low bioavailability of resveratrol and consequently its effectiveness. In view of the results obtained in the present study, it can be proposed that the combination of quercetin and resveratrol could be more effective, in terms of body fat reduction, than the administration of these molecules separately. This proposal is justified not only by the effects on resveratrol bioavailability, as proposed in the literature, but also by the fact that at 1 *μ*M (a dose close to quercetin and resveratrol serum concentrations found in* in vivo* studies) quercetin is more effective in reducing adipogenesis in preadipocytes whereas resveratrol is more effective in inhibiting lipid metabolism in mature adipocytes. Thus, the combination of these two molecules can induce body fat reduction more effectively by targeting both cell types, preadipocytes and adipocytes, at the same time.

Taking all the data presented into account, it can be concluded that doses of quercetin in the range of serum concentrations are able to inhibit adipogenesis, but higher doses, at least 10 *μ*M, are needed to reduce fat accumulation in mature adipocytes. In the case of maturing preadipocytes, 1 *μ*M of quercetin exerts its antiadipogenic effect at the early stages of adipogenesis, whereas 10 *μ*M of quercetin acts at the later stages.

## Figures and Tables

**Table 1:** Primers for PCR amplification of each studied gene.

	Sense primer	Antisense primer	Probe
SYBR Green RT-PCR:			
LPL	5′-CAG CTG GGC CTAACT TTG AG-3′	5′-CCT CTC TGC AAT CAC ACG AA-3′	
PPAR*γ*	5′-ATT CTG GCC CAC CAA CTT CGG-3′	5′-TGG AAG CCT GAT GCT TTA TCC CCA-3′	
SREBP1c	5′-AAA TCT TGC TGC CAT TCG-3′	5′-TTG ATC CCG GAA GCT CTG TG-3′	
CEBP*α*	5′-TTC CTC CGG CTA AGA CTT AGG C-3′	5′-CAG GGG TGT GTG TAT GAA CTG G-3′	
ATGL	5′-CAC TTT AGC TCC AAG GAT GA-3′	5′-TGG TTC AGT AGG CCA TTC CT-3′	
HSL	5′-GGT GAC ACT CGC AGA AGA CAA TA-3′	5′-GCC GCC GTG CTG TCT CT-3′	
FASN	5′-AGC CCC TCA AGT GCA CAG TG-3′	5′-TGC CAA TGT GTT TTC CCT GA-3′	
ACC	5′-GGA CCA CTG CAT GGA ATG TTA A-3′	5′-TGA GTG ACT GCC GAA ACA TCT C-3′	
PGC1*α*	5′-CCA AAG CTG AAG CCC TCT TGC-3′	5′-GTT TAG TCT TCC TTT CCT CGT GTC C-3′	
Leptin	5′-TGG ACC AGA CTC TGG CAG TC-3′	5′-AGG ACA CCA TCC AGG CTC TC-3′	
Adiponectin	5′-TG TAG GAT TGT CAG TGG ATC TG-3′	5′-GCT CTT CAG TTG TAG TAA CGT CAT C-3′	
Apelin	5′-ATT TAA GGA CAC GCT GAT CAA AGG-3′	5′-AGT CCC GAA AGT ATT CAA AAG CAG-3′	
*β*-actin	5′-ACG AGG CCC AGA GCA AGA G-3′	5′-GGT GTG GTG CCA GAT CTT CTC-3′	
TaqMan RT-PCR:			
CEBP*β*	5′-GAG CGA CGA GTA CAA GAT GCG-3′	5′-GCT GCT CCA CCT TCT TCT GC-3′	5′-FAM-TCG TTC TCC GCC GTC AGC TCC AGC-TAMRA-3′
SIRT-1	5′-GAC GAC GAG GGC GAG GAG-3′	5′-ACA GGA GGT TGT CTC GGT AGC-3′	5′-FAM-CTG CCG CCG CCG CTG CCG-TAMRA-3′
Visfatin	5′-CCG GCC CGA GAT GAA TGC-3′	5′-GGA ATA AAC TTT GCT TGT GTT GGG-3′	5′-FAM-AGC CGA GTT CAA CAT CCT GCT GGC-TAMRA-3′
β-actin	5′-TCT ATG AGG GCT ACG CTC TCC-3′	5′-CAC GCT CGG TCA GGA TCT TC-3′	5′-FAM-CCT GCG TCT GGA CCT GGC TGG C-TAMRA-3′

LPL = lipoprotein lipase, PPARγ = peroxisome proliferator-activated receptor, SREBP = sterol regulatory element-binding protein, C/EBPα and C/EBPβ = CCAAT-enhancer-binding proteins alpha and beta, ATGL = adipose triglyceride lipase, HSL = hormone sensitive lipase, FASN = fatty acid synthase, ACC = acetyl-CoA carboxylase, PGC-1 *α* = peroxisome proliferator-activated receptor gamma coactivator, and SIRT1 = deacetylase sirtuin 1.

**Table 2:** Relative triacylglycerol content (arbitrary units) after quercetin treatment in maturing preadipocytes and mature adipocytes.

	C	Q0.1	*P*	Q0.5	*P*	Q1	*P*	Q2	*P*	Q5	*P*	Q10	*P*
Stage of adipogenesis													
Whole adipogenesis (d0–d8)	100 ± 5.1	77.95 ± 12.8	0.09	75.57 ± 8.6	<0.01	58.4 ± 8.6	<0.001	81.3 ± 7.4	<0.05	79.6 ± 7.2	<0.05	62.9 ± 10.2	<0.01
Early stage (day 0–60 hours)	100 ± 6.9					86.0 ± 5.7	0.09					95.3 ± 7.3	ND
Late stage (60 hours–d8)						92.2 ± 5.6	ND					75.9 ± 10.5	<0.05
Mature adipocytes (d12 for 24 h)	100 ± 6.3					109.3 ± 34.0	ND	86.2 ± 3.4	0.07	79.3 ± 12.8	0.10	76.3 ± 4.8	<0.01

C = control; ND = no difference, Q0.1 = 0.1 *µ*M of quercetin, Q0.5 = 0.5 *µ*M of quercetin, Q1 = 1 *µ*M of quercetin, Q2 = 2 *µ*M of quercetin, Q5 = 5 *µ*M of quercetin, and Q10 = 10 *µ*M of quercetin.

Values are means ± SEM. Comparisons between each treatment and the controls were analyzed by Student's *t*-test. The difference was established at *P* ≤ 0.10.
